# Supplementary material for: Perspectives of healthcare professionals in England on falls interventions for people with dementia: a qualitative interview study
Source: BMJ Open. 2019 Feb 11;9(2):e025702. doi: 10.1136/bmjopen-2018-025702 (PMC6377506; doi:10.1136/bmjopen-2018-025702)
Supplement: Supplementary file 1 [file bmjopen-2018-025702supp001.pdf]

Appendix A – Interview schedule for participants working in the Falls Service

1. Introduction: Explanation of research aims, nature and purpose of interview: The aim of this study is to develop an intervention to help prevent falls among patients with early cognitive impairment including dementia. I would like to find out about your experience of supporting such patients, and your views about what kind of interventions would be effective and acceptable to patients and their carers.

2. Discussion of concerns/questions about the interview/research, confirmation of confidentiality, voluntary nature of participation, that respondent may withdraw at any time, willingness to take part in the interview, acceptance (or otherwise) of the interview being recorded, and obtaining signed consent.

3. What are the main reasons for patients to be referred to the falls clinic?
    a. Where do the referrals come from?
    b. What level/duration of support is available?

4. How many of your patients are affected by dementia/cognitive impairment?
    a. Establish if case load is mainly with early stage or wider spectrum of severity

5. Could you tell me about a recent or current patient with early dementia or cognitive impairment and the kind of interventions you were/are using to help prevent them fall?

6. How do you think patients with early dementia or cognitive impairment feel about falling?
    a. Apprehensive, unconcerned, prefer to conceal etc
    b. What kind of strategies do you think they might use to help stop themselves from falling?

7. Do you always know if a patient is affected by dementia?
    a. Do you ever think that a patient might have early dementia which has not been diagnosed?
    b. What would make you suspect this was the case?

8. What are the main reasons why people with early dementia fall?
    a. Any differences for people without dementia?

9. What kind of interventions and techniques do you currently use to prevent people with early dementia falling?

    a. What is the rationale for each of these?
    b. Standard interventions, or tailored to each case?
    c. Assessment of effectiveness of each one mentioned
    d. Differences between patients with and without dementia?

   e. Grounded in physical techniques and strategies, or cognitive involvement also?

10. Are there any particular obstacles or difficulties you encounter in working with this group of patients?
    a. Motivation, denial, non-comprehension, non-compliance etc

11. What do you think would be the most effective interventions to stop early dementia patients from falling?
    a. How feasible are these?
    b. How acceptable to patients and carers?

Note – establish what R thinks about effective interventions before presenting R with the following list to consider

12. How useful/feasible do you think each of the following possibilities would be:
    i. Doing exercises at home
    ii. Having a falls check up,
        1. at home
        2. in a clinic
    iii. Attending a clinic and taking part in activities there
    iv. Using a stick or other support aids.
    v. Being told to avoid things that might increase risk, such as doing things in a hurry or doing two things at once
    vi. Practicing mental exercises
    vii. Learning new ways of doing everyday activities, such as talking out loud while doing a task
    viii. Anything else you can think of that might be helpful?

13. Do you think it is possible to stop people with early dementia from falling?

14. In terms of complexity of intervention, and intensity of effort and application, what do you think it is reasonable to ask patients to do? (eg re specific types of intervention, or in combination)?
    a. Are there any problems you would anticipate in implementing such interventions?

15. How do you think the work of the falls clinic relates to the kind of support offered by memory clinics?
    a. E.g. roles being similar, overlapping, complementary, entirely separate
    b. Any scope for closer integration between the two services in supporting dementia patients?

16. Conclusion

    Thank you very much for your time and help with the study.

    Are there any other issues relating to falls you think are important, that we haven't talked about?

    Are there any questions you would like to ask me about the study?

    Thanks and close. Offer to send summary of findings when study is finished.

Appendix B – Interview schedule for participants working in the Memory Clinic

1. Introduction: Explanation of research aims, nature and purpose of interview: The aim of this study is to develop an intervention to help prevent falls among patients with early cognitive impairment including dementia. I would like to find out about your experience of supporting such patients, and your views about what kind of interventions would be effective and acceptable to patients and their carers.

2. Discussion of concerns/questions about the interview/research, confirmation of confidentiality, voluntary nature of participation, that respondent may withdraw at any time, willingness to take part in the interview, acceptance (or otherwise) of the interview being recorded, and obtaining signed consent.

3. How do patients get referred to the memory clinic?
    a. Where do the referrals come from?
    b. What level/duration of support is available?

4. How many of your patients are affected by *early* dementia/cognitive impairment?
    a. Establish if case load is mainly with early stage or wider spectrum of severity

5. Could you tell me about a recent or current patient with early dementia and the kind of interventions you were/are using to improve their memory?

6. How do you think patients with early dementia respond to their memory problems?
    a. Apprehensive, unconcerned, prefer to conceal etc
    b. What kind of strategies do you think they might use to reduce the impact of these?

7. What kind of interventions and techniques do you currently use to help early dementia patients improve their memory?
    a. What is the rationale for each of these?
    b. Standard interventions, or tailored to each case?
    c. Assessment of effectiveness of each one mentioned
    d. Grounded in physical techniques and strategies (e.g. notes, placing, specific placing of objects ??), or cognitive involvement also (mneumonics, rehearsal ??)?

8. Are there any particular obstacles or difficulties you encounter in working with this group of patients?
    a. Motivation, denial, non-comprehension, non-compliance etc

9. Do you find that falling is an issue for patients with early dementia?
    a. How often, how serious.

10. If a patient is at risk, or has a history of falling, are there any specific interventions you would use to try and help them prevent falls?
    a. What techniques? Rationale? How effective?
    b. Or do you think that preventing falls is not within the remit of the memory clinic?

11. Are there any other things that you/the memory clinic could do to reduce the incidence of falls among early dementia patients?

Note – establish what R thinks about the effectiveness of interventions before presenting R with the following list to consider

12. How useful/feasible do you think each of the following possibilities would be:
    i. Doing exercises at home
    ii. Having a falls check up,
        1. at home
        2. in a clinic
    iii. Attending a clinic and taking part in activities there
    iv. Using a stick or other support aids.
    v. Being told to avoid things that might increase risk, such as doing things in a hurry or doing two things at once
    vi. Practicing mental exercises
    vii. Learning new ways of doing everyday activities, such as talking out loud while doing a task
    viii. Anything else you can think of that might be helpful?

13. Do you think it is possible to stop people with early dementia from falling?

14. In terms of complexity of intervention, and intensity of effort and application, what do you think it is reasonable to ask patients to do? (eg re specific types of intervention, or in combination)?
    a. Are there any problems you would anticipate in implementing such interventions?

15. How do you think the work of the memory clinic relates to the kind of support offered by falls clinics?
    a. E.g. roles being similar, overlapping, complementary, entirely separate
    b. Any scope for closer integration between the two services in supporting dementia patients?

16. Conclusion
    Thank you very much for your time and help with the study.
    Are there any other issues relating to falls you think are important, that we haven't talked about?
    Are there any questions you would like to ask me about the study?
    Thanks and close. Offer to send summary of findings when study is finished.
